# Comparison of accuracy and speed between plaster casting, high‐cost and low‐cost 3D scanners to capture foot, ankle and lower leg morphology of children requiring ankle‐foot orthoses

**DOI:** 10.1002/jfa2.70006

**Published:** 2024-08-27

**Authors:** Muhannad Farhan, Joyce Zhanzi Wang, Rachael Warncke, Tegan Laura Cheng, Joshua Burns

**Affiliations:** ^1^ University of Sydney School of Health Sciences & Children's Hospital at Westmead Sydney New South Wales Australia; ^2^ Faculty of Medical Rehabilitation Science Taibah University Al Madinah Al Munawarah Madinah Saudi Arabia; ^3^ Engineering Prototypes & Implants for Children (EPIC) Lab The Children's Hospital at Westmead Sydney New South Wales Australia; ^4^ Orthotics Department The Children's Hospital at Westmead Sydney New South Wales Australia; ^5^ St. Jude Children's Research Hospital Memphis Tennessee USA

**Keywords:** 3D scanner, accuracy, ankle foot orthosis, foot measurement

## Abstract

**Background:**

Traditional plaster‐cast fabrication of an ankle‐foot orthosis (AFO), although robust, is time‐consuming and cumbersome. 3D scanning is quickly gaining attention as an alternative to plaster casting the foot and ankle region for AFO fabrication. The aim of this study was to assess the accuracy and speed of two high‐performing 3D scanners compared with plaster casting in pediatric patients requiring an AFO.

**Methods:**

Ten participants (mean age 10.0 ± 3.9 years) prescribed AFOs for a movement disorder were 3D scanned with the high‐cost Artec Eva (Eva) and low‐cost Structure Sensor II (SSII) using one‐person (1p) and two‐person (2p) protocols. Accuracy and speed for both 3D scanners were compared with corresponding plaster cast measures (≤5% acceptable difference). Bland and Altman plots were generated to show mean bias and limits of agreement.

**Results:**

Overall, Eva and SSII were accurate for foot, ankle, and lower leg key clinical landmarks (Eva‐1p: 4.4 ± 7.3%; Eva‐2p: 3.2 ± 7.5%; SSII‐1p: 0.6 ± 7.4%; SSII‐2p: 0.7 ± 8.2%). Bland and Altman plots for the SSII demonstrated lower biases for 1p (bias 0.5 mm, LoA: −12.4–13.5 mm) and 2p (0.4 mm, LoA: −11.4–12.2 mm) protocols compared with Eva for 1p (bias 2.3 mm, LoA: −8.0–12.7 mm) and 2p (1.8 mm, LoA: −10.7–14.3 mm) protocols. The SSII 2p protocol was the fastest 3D scanning method (26.4 ± 11.1 s).

**Conclusions:**

The high‐cost Eva and low‐cost SSII 3D scanners using the 1p and 2p protocols produced comparable accuracy and faster capture of key clinical landmarks compared with plaster cast measures for the fabrication of AFOs in pediatric patients.

## 1 INTRODUCTION

Ankle‐foot orthoses (AFOs) are externally worn assistive devices that are prescribed for the accommodation and correction of foot and ankle pathologies causing impaired gait and posture. In children with movement disorders such as Charcot–Marie–Tooth disease (CMT) and cerebral palsy (CP), AFOs are commonly prescribed for foot drop [1]. An individual with foot drop is unable to appropriately position and dorsiflex their ankle during the swing phase and loading response of the gait cycle, resulting in inefficient compensations at the knee and hip and/or trips and falls. To correct this, an ankle‐foot orthosis (AFO) supports the ankle joint and foot to assist in dorsiflexion and restricts excessive plantarflexion during terminal stance and swing phases.

The traditional method of AFO fabrication begins with plaster casting of the foot, ankle and lower leg, is dependent on clinician expertise and purpose‐built workspaces, can be time consuming and generates significant waste material [2]. The plaster cast is just the first step of the laborious manufacturing process for traditional AFOs, which can lead to long waiting lists and delay the delivery of necessary assistive devices. Specifically for children, long waiting times for traditional AFOs are impractical due to rapid periods of growth and development [3, 4]. AFO users and their caregivers have reported their frustrations with the lengthy process of fabrication for traditional devices, including the high number of clinic visits that interfere with their child's routine and school activities [5]. Further, traditional plaster casting can prove difficult to position children for the time required for the plaster to be applied and dry sufficiently. Parents and caregivers have requested an alternative and more efficient method for AFO production, anticipating the introduction of digital fabrication. Digital fabrication methods, which involves the use of 3D scanners to capture limb morphology and 3D printing to manufacture the devices, has the potential to offer more efficient, as well as comparable or more effective AFOs [6, 7]. In a study of 12 children with CMT, 3D printed AFOs fabricated from 3D scanning the plaster cast of the participant's lower legs improved gait compared to traditionally fabricated AFOs, with the 3D printed AFOs also being lighter in weight [8]. Another study comparing the gait of seven participants (including children with CP) with digitally fabricated AFOs, based on scanning the lower leg directly or scanning the plaster cast against traditionally manufactured AFOs, found a comparable improvement in gait [9]. However, a randomised controlled trial found that laser 3D scanning did not improve the quality or speed of AFO delivery compared to plaster casting, primarily due to the need for remakes and rescans of the 3D scanned group [10]. An accurate and reliable 3D scan of the foot, ankle and lower limb is clearly a critical aspect of an effective digital AFO workflow.

There are a range of commercially available 3D scanners and technologies (e.g., structured light, laser, photogrammetry) that can capture the morphology of the patient's foot, ankle and lower leg, and then convert it into a digital form. These 3D scanners vary in accuracy, speed and cost [6]. Cost may be a deciding factor for orthotists, podiatrists and other healthcare professionals deciding which 3D scanner to purchase, especially in relation to 3D scan quality. Several studies have shown that low‐cost 3D scanners (<$1000) can reliably and accurately capture key clinical characteristics of the face, abdomen and foot [11, 12, 13, 14]. However, in a study comparing the high‐cost Artec Eva and the low‐cost iSense for capturing knee morphology in 14 limbs to fabricate 3D printed braces, both scanners overestimated leg circumference, with the iSense more so than the Eva [15].

There have been no studies that have compared high‐cost and low‐cost 3D scanners for capturing key clinical landmarks of the foot, ankle and lower leg region for AFO fabrication and how these compare with plaster casting. Further, while plaster casting usually requires a single practitioner, 3D scanning while manually correcting malalignment of the lower leg may require an additional person to hold the 3D scanner and produce a viable 3D scan. Therefore, this study sought to address the research gaps in exploring and comparing multiple 3D scanner price points with traditional plaster casting.

## 2 MATERIALS AND METHODS

### 2.1 Aim of the study

The aim of this study was to assess the accuracy and speed of two 3D scanners (high‐cost Artec Eva and low‐cost Structure Sensor II) for capturing the foot, ankle and lower leg of children prescribed with AFOs using a one‐person and two‐person scanning protocol as compared with traditional plaster casting.

### 2.2 Design of the study

Repeated‐measures design.

### 2.3 Setting

The study was conducted alongside a regular appointment with a treating orthotist for an AFO plaster casting in the Orthotics Department of a public tertiary children's hospital through consecutive sampling method. Potential participants and their families were sent an invitation letter and information sheet about the study protocol prior to their appointment. If the participant/guardian agreed to take part in the study at their appointment, they were screened for eligibility. The inclusion criteria for this study were pediatric patients with a movement disorder affecting gait attending the hospital for an AFO to improve walking ability. A participant was excluded if they were prescribed any other assistive device to aid ambulation, if the participant or their parent/guardian had trouble communicating in English and required a translator, if the participant had a history of epilepsy or seizures when targeted by a flashing light or if the participant was non‐ambulatory. If the participant and/or their guardian agreed to take part in the study, informed written consent was obtained on the day of their appointment.

Once informed consent was obtained, one of the investigators (MF) would sit in on the participant's appointment to time the plaster casting process. Plaster casting was conducted by a qualified orthotist from the hospital's Orthotics Department with experience in producing AFOs for children as part of their regular practice. The participant was cast conventionally in a seated position with their leg hanging over the side of the examination table with their lower leg positioned by the orthotist as per the clinical requirements. Following the clinical session, the research session began. If the participant did not have time following the clinical appointment, they took part in the research session when they returned for their AFO fitting session some weeks later (this only occurred for one participant).

### 2.4 Equipment

**FIGURE 1:**
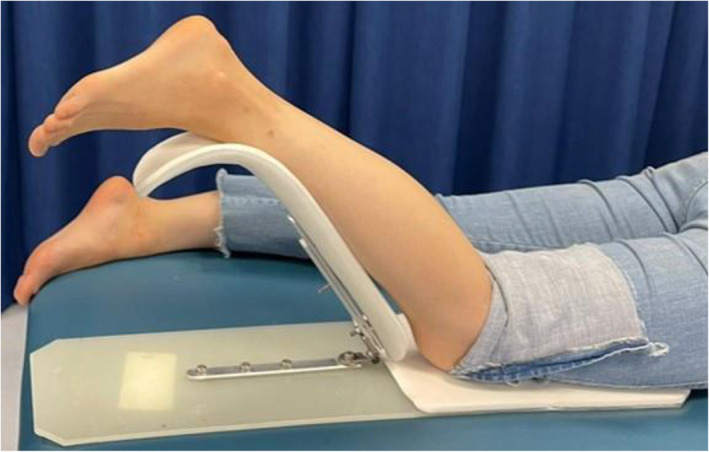
Custom‐made 3D scanning jig (scan stand) to position the participant's leg during the 3D scanning process.

Two scanners were selected based on preliminary testing of reliability, accuracy, speed and availability from an earlier study of seven 3D scanners [16]: Artec Eva (Eva; Artec 3D, Luxembourg) and Structure Sensor II (SSII; Occipital, CO, USA). At the time of purchase, the cost of the Eva was AUD$19,800 (requires a laptop costing approx. AUD$3000) and the cost of the SSII was AUD$622 (requires iPad costing approx. AUD$550). Both 3D scanners use structured light technology, with the Eva using white light projection and the SSII using infrared light projection to capture limb morphology. To minimise involuntary leg movements during scanning, a bespoke 3D scanning jig (called the Scan Stand) was used to support the limb for all protocols, see Figure 1.

### 2.5 Participant assessment

Assessment data were collected to see if factors such as condition, deformity and appearance of participants would influence the success of the 3D scanning protocol. A standard assessment form was developed to collect demographics and physical characteristics including age, sex, medical condition, AFO type and goal, foot alignment (Foot Posture Index, FPI) and skin tone. Skin tone was determined by comparing a close‐up photo of the participant's leg against a prosthetics silicone color swatch (Skinergy Plus), which included 19 different color tones ranging from E0 (light) to E19 (very dark). The FPI is a well‐validated measure of foot alignment ranging from −12 for highly cavovarus to +12 for highly planovalgus foot alignment [17].

### 2.6 Key clinical landmarks

Key foot, ankle and lower leg clinical landmarks necessary for AFO fabrication were measured directly from each patient (clinical) and from their plaster cast. Table 1 describes the measurement points of the key clinical landmarks assessed in this study. Key clinical landmarks were identified by placing reflective markers (3 mm hemisphere markers; Vicon Motion Systems Ltd, UK) on the relevant bony prominences of the participant's foot, ankle and lower leg. For patient and cast measures, the key clinical landmarks were measured manually using digital calipers, manual calipers and a ruler. Since clinical measures mirrored cast measures (1.93% ± 4.87% difference), the cast measures were treated as the reference standard for comparison with 3D scanning protocols since casting is the most widely used method for AFO fabrication.

**TABLE 1:** Key clinical foot, ankle and lower leg landmarks measured directly and of the cast.

Parameter	Measurement
Forefoot	Distance between markers placed on the center of the medial side of the first metatarsal head and lateral side of the fifth metatarsal head.
Rearfoot	Distance between markers placed on the medial and lateral sides of the calcaneus at 21% of the foot length, based on average clinical and cadaveric proportions.
Medial arch height	Vertical distance between a marker placed at the navicular tuberosity and a horizontal line with the plantar surface of the foot.
Malleolus width	Distance between markers placed on the center of the lateral and medial malleoli.
Midcalf width	Distance between markers placed on the medial and lateral sides of the calf 3 cm below fibula head.
Foot length	Distance from the most distal aspect of the longest toe to the most proximal aspect of the calcaneus.

### 2.7 3D scanning protocols

Each participant was scanned by two 3D scanners (Eva and SSII) on an examination table laying in a prone position. Their knee was flexed at approximately 45° and supported at the shin, foot and ankle by a bespoke 3D scanning jig. There were two 3D scanning protocols for each 3D scanner: (i) one‐person protocol (1p) and (ii) two‐person protocol (2p) (see Supplementary Tables S1–S4). The rationale behind these two protocols was to understand if a child's leg supported by a 3D scanning jig would be sufficient or whether an additional clinician would be required to provide a ‘helping hand’. For the 1p protocol, the investigator would position the participant's leg on the jig and operate the 3D scanner. For the 2p protocol, an investigator would hold the participant's foot in a clinically appropriate position, and the second investigator would operate the 3D scanner. The intent of the two protocols was to understand whether a range of patient skin tone or foot deformities could be captured by the 1p protocol or whether in some cases a 2p protocol is necessary. Hence, there were four 3D scanning trials in this study: Eva 1p, Eva 2p, SSII 1p and SSII 2p. These four protocols were carried out in this order for each participant. Depending on the protocol, one of two investigators (MF, JW) operated the 3D scanner, both with ∼3 years of experience in 3D scanning the lower leg.

### 2.8 Outcome measures

*Accuracy*. Mean difference of the foot, ankle and lower leg key clinical landmarks between the four 3D scanning protocols compared with cast measures was the primary outcome measure. For 3D scan measures, 3‐Matics (version 14.0; Materialise NA) software was used. The dimensions of each parameter were measured using the point‐to‐point ‘*measure distance*’ tool between the reflective markers. A datum plate was aligned with the plantar surface of the mesh to measure arch height.

*Speed*. The secondary outcome measure was the time taken using a stopwatch to be cast or 3D scanned with each protocol. The starting point of the plaster casting was noted when the technician started applying the plaster bandage to the foot, ankle and lower leg. The end point was noted when the clinician removed the plaster cast from the patient's leg. The plaster time did not include plaster preparation or drying time beyond removal from the leg. The 3D scanning process was timed from the pressing of the start button and finished by pressing the stop button. The 3D scanning time did not include any post‐processing time. Although the additional times associated with plaster cast tidying, drying, positive cast production and 3D scanning post‐processing are of interest they were outside the focus of this study.

### 2.9 Analyses

One limb only from each participant was included in this 3D scanning study to satisfy the independence requirement for statistical analysis. For participants requiring bilateral AFOs, the dominant side was chosen for scanning, and for those prescribed a unilateral AFO, the affected side was chosen. A formal power analysis was not performed for this cross‐sectional observational study because recruitment was limited to available patients attending the Orthotics Department during the study window which was interrupted by the COVID‐19 pandemic.

The accuracy of digital mesh measurements for each 3D scan was compared with the corresponding cast measures in two ways: by percentage difference and by Bland and Altman plots. Percentage difference as a non‐dimensional unit was calculated across key clinical landmarks to normalise the different sizes of participants and range of measurements. The percentage difference was calculated by subtracting the cast measure from the 3D scan measure, divided by the cast measure. The percentage difference was calculated for each foot, ankle and lower leg parameter for each participant. The average of all key clinical landmark measured was presented as an overall mean and standard deviation (SD), as well as for each parameter. If the mesh measures were larger than the cast measures, the data point was plotted as being in the positive *y* axis and vice versa. A percentage difference of 5% was set as an acceptable difference for a clinical environment, that is, 95% or better agreement. While a 5% difference can represent errors of different absolute magnitudes, we felt that this was an intuitive and conservative approach to help clinicians and scientists interpret the error of measurement. In addition to the 5% error threshold, differences in raw values (mm) are provided in Supplementary Table S5, and Bland and Altman plots showing the level of agreement and the mean bias for each 3D scanner and measurement parameter are provided in Supplementary Figures S1–S6. Bland and Altman plots were constructed to determine the overall and parameter‐level accuracy for each 3D scanning protocol. Bland and Altman plots graph the difference between measures obtained by two methods against the average of the measures [18]. In each graph, three horizontal lines were marked:

1. A solid line, called the bias, showing the overall mean difference between 3D scanner measures and the cast measures.
2. Two dashed horizontal lines, called the limit of agreement (LoA). These lines are plotted by taking ±2SD of the overall difference between 3D scan measures and cast measures [19, 20].

In addition, a simple linear regression was applied for the data points to examine correlation and is represented by a blue solid line in the plots, with blue dotted lines showing 95% confidence bands.

## 3 RESULTS

### 3.1 Participant demographics

**FIGURE 2:**
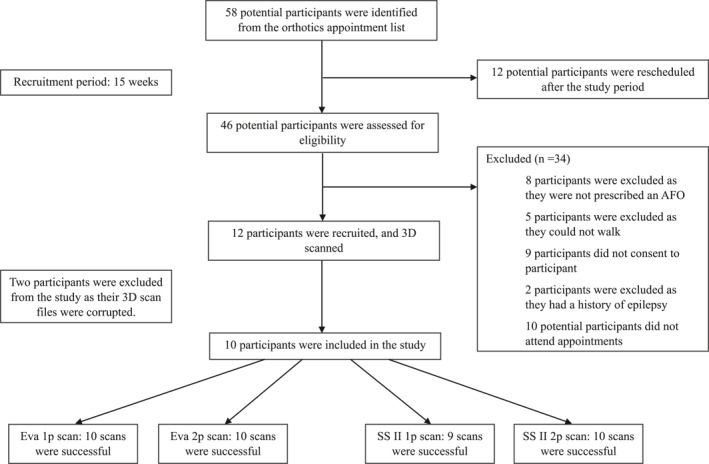
Flow chart showing participant recruitment for the study.

A total of 58 potential participants were screened for this study and sent letters of invitation. Of these, 12 participants were unavailable because they had to reschedule their appointments to after the study period and 34 participants were excluded due to ineligibility (Figure 2). Twelve participants volunteered to take part in this study; however, the 3D scan files of two participants were corrupted and these were excluded from analysis.

Ten participants were included in the analysis, with their demographic and physical characteristics summarised in Table 2. Participant skin tone ranged from E2 to E8, which clustered the participants on the paler end of the scale. Participant foot posture index ranged from −7 (moderate cavovarus) to +12 (severe planovalgus).

**TABLE 2:** Demographics and physical characteristics of 10 participants included in the study.

Age (years)	Sex	Diagnosis	AFO goals	Skin tone	Foot posture index	AFO prescription
10	Female	Charcot‐Marie‐Tooth disease	Support foot and ankle during walking	4	−7	Leaf spring with SMO insert
13	Male	LMNA‐related congenital muscular dystrophy	Support and contracture management	4	0	Solid
18	Male	Diplegic cerebral palsy	Improve walking and manage ankle contracture	2	0	Solid
14	Male	Hemiplegic cerebral palsy	Improve walking and manage ankle contracture	3	9	Solid
6	Male	Hemiplegic cerebral palsy	Improve balance and walking	5	1	Hinged
9	Male	Hemiplegic cerebral palsy	Improve walking and ankle range	6	2	Hinged
9	Male	Fibular Hemimelia	Walking better after surgery	4	12	Solid
6	Female	Idiopathic toe walking	Increase ankle dorsiflexion range	8	3	Hinged
6	Male	Idiopathic toe walking	Improve walking and stop toe walking	7	1	Solid
9	Male	Bilateral Clubfoot	Improve walking and ankle range	5	−3	Hinged

### 3.2 Quality of 3D scanning protocols

**FIGURE 3:**
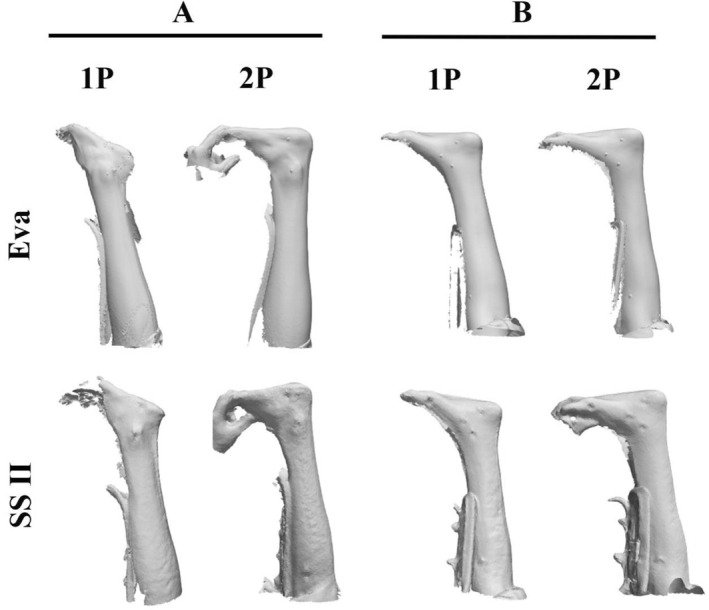
Examples of 3D scans from two participants using the Artec Eva (Eva) and structure sensor mark II (SSII). (A). This participant was successfully captured by both the Eva 1p and 2p and SSII 2p protocols, but unsuccessfully captured by the SSII 1p protocol. (B). A participant that was successfully 3D scanned with all protocols.

A 3D scan was considered successful if all foot, ankle and lower leg reflective markers were captured and digital key clinical landmark could be calculated. For the Eva, both 1p and 2p scanning protocols produced acceptable 3D scans for all participants. The SSII 2p protocol was also acceptable for all participants, while the SSII 1p protocol was acceptable for nine participants. One scan with the SSII 1p protocol was unsuccessful because of participant movement (Figure 3). The measurements for this unsuccessful 3D scan are included in the analyses, apart from forefoot, rearfoot, foot length and arch height key clinical landmark which could not be measured.

### 3.3 Accuracy

**FIGURE 4:**
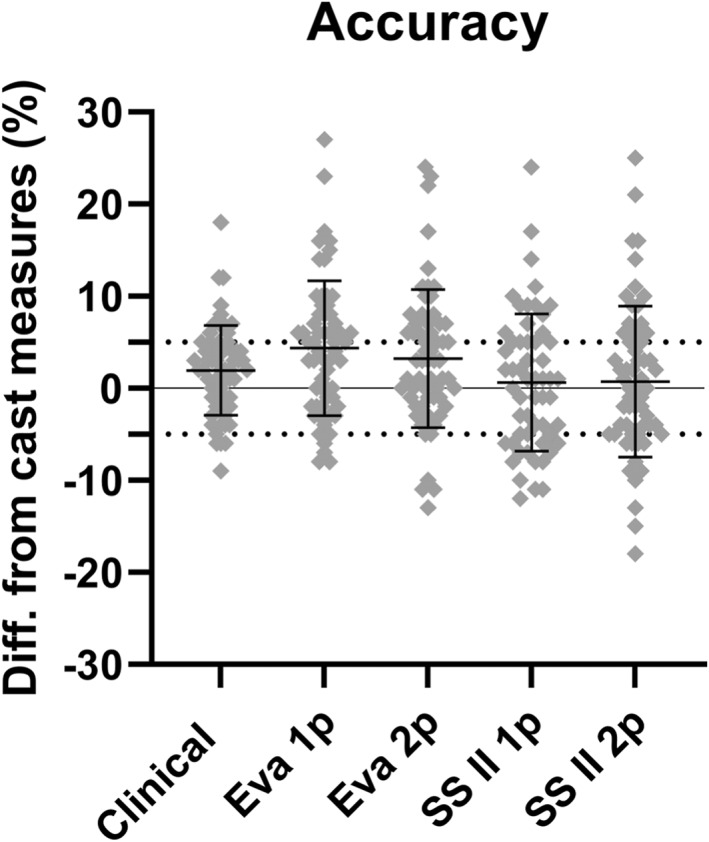
Overall percentage difference between 3D scan and cast measures of the foot, ankle and lower leg. The 3D scanning protocols are Artec Eva with one person (Eva 1p), Artec Eva with two persons (Eva 2p), Structure Sensor II with one person (SSII 1p), and Structure Sensor II with two persons (SSII 2p). Positive differences in the *y*‐axis show that the 3D scan was larger in size than the cast measure. Dotted line represents 5% difference.

Figure 4 depicts the percentage difference between 3D scans and plaster cast measures. For both the Eva and SSII, the mean percentage differences were within the acceptable range, that is, ≤5% difference. The mean ± SD percentage difference for Eva 1p was 4.35 ± 7.31%, Eva 2p was 3.23% ± 7.51%, SSII 1p was 0.62% ± 7.45% and SSII 2p was 0.73% ± 8.20%.

**FIGURE 5:**
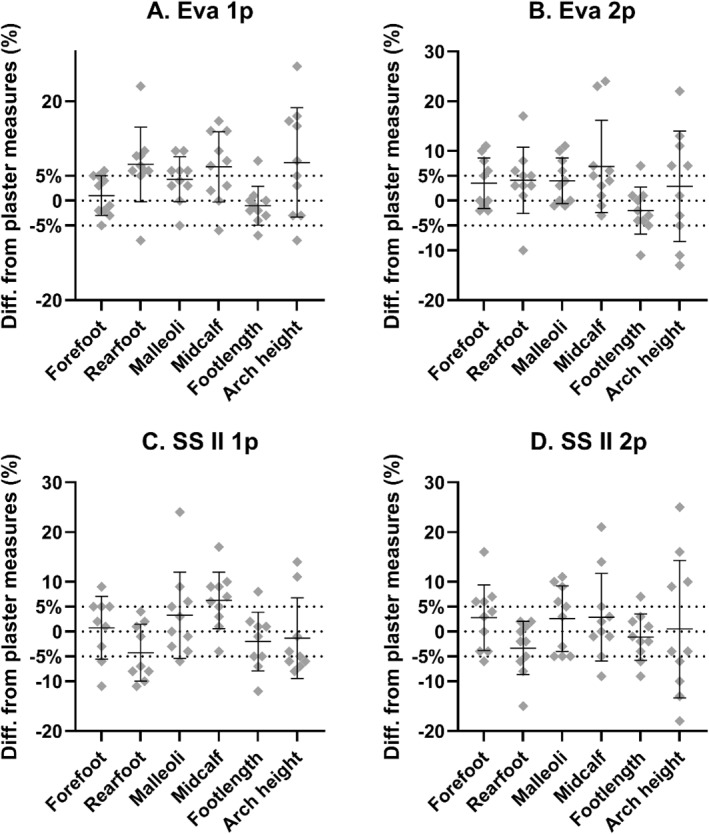
Key clinical landmark percentage differences between 3D scans and cast measures. The 3D scanning protocols are (A). Eva with one person (Eva 1p), (B). Artec Eva with two persons (Eva 2p), (C). Structure Sensor II with one person (SSII 1p), and D. Structure Sensor II with two persons (SSII 2p). Positive differences in the *y*‐axis show that the 3D scan was larger in size than the plaster cast measurement. Dotted line represents 5% difference.

At foot, ankle and lower leg key clinical landmarks, the Eva 1p protocol was within an acceptable range for forefoot width (1.0% ± 4.0), malleoli width (4.3% ± 4.5%) and foot length (−1.0% ± 3.9%; Figure 5). The Eva 2p protocol was within the acceptable range for all key clinical landmarks except midcalf length (6.9% ± 9.2%). The SSII 1p protocol was within the acceptable range for all key clinical landmarker except midcalf length (6.3% ± 5.6%), while the SSII 2p protocol was within the acceptable range for all six key clinical landmarks (Figure 5).

**FIGURE 6:**
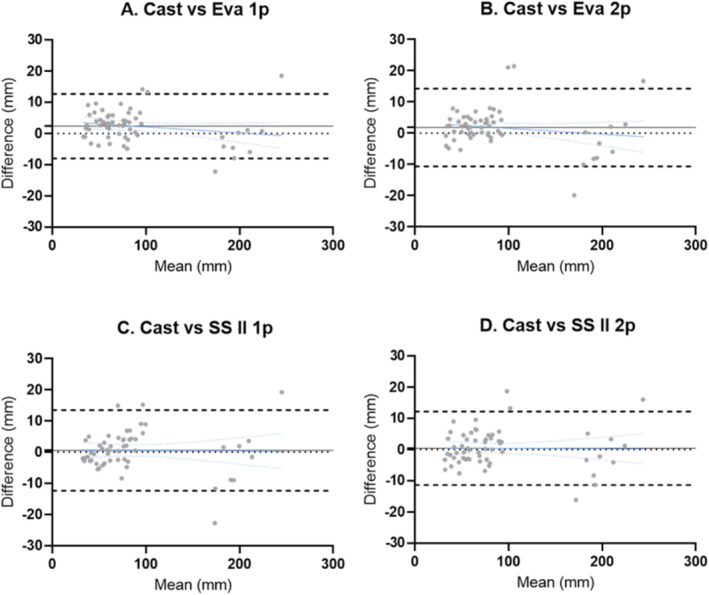
Bland and Altman plots illustrating the overall agreement between 3D scan and cast measures of the foot, ankle and lower leg. The 3D scanning protocols are (A). Eva with one person (Eva 1p), (B). Artec Eva with two persons (Eva 2p), (C). Structure Sensor II with one person (SSII 1p), and (D). Structure Sensor II with two persons (SSII 2p). Each plot presents a dotted line representing zero, a solid line representing the mean bias, two dashed lines representing the upper and lower limits of agreement (LoA), and a blue line representing simple linear regression and blue dotted line for 95% confidence bands in the plots.

Figure 6 shows the Bland and Altman plots for all measures in absolute units (mm) and upper/lower levels of agreement (LoA) for each 3D scanner mesh compared with cast measures. The Eva showed higher mean biases for the 1p protocol (bias: 2.30 mm, LoA: −7.96–12.72 mm) and 2p protocol (1.80 mm, LoA: −10.66–14.25 mm) compared to the SSII 1p protocol (bias: 0.51 mm; LoA: −12.45–13.48 mm) and 2p protocol (bias: 0.43 mm; LoA: −11.37–12.24 mm).

Bland and Altman plots at the foot, ankle and lower leg showed similar trends as the overall accuracy analysis (see Supplementary Figures S1–S6, in Additional File 2). The SSII 2p protocol showed the smallest mean bias for four out of the six key clinical landmarks, that is, rearfoot (1.49 mm, LoA: −7.05–3.60 mm), malleoli width (1.49 mm, LoA: −6.53–9.51 mm), midcalf width (2.74 mm, LoA: −12.47–17.96 mm), and arch height (0.193 mm, LoA: −9.90–10.29 mm). For foot length, Eva 1p protocol had the lowest mean bias (−1.53 mm, LoA: −17.64–14.57 mm), while for forefoot width SSII 1p protocol exhibited the lowest mean bias (0.63 mm, −9.05–10.32 mm).

### 3.4 Speed

**FIGURE 7:**
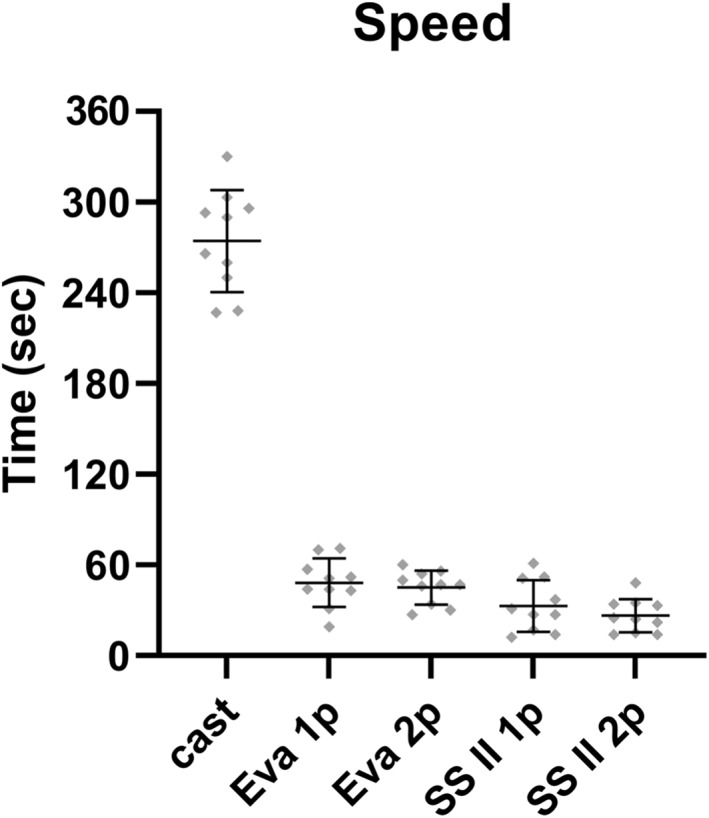
Time taken to obtain a complete 3D scan of the foot, ankle and lower leg by 3D scan protocols and plaster casting. The 3D scanning protocols are Artec Eva with one person (Eva 1p), Artec Eva with two persons (Eva 1p), Structure Sensor II with one person (SSII 1p) and Structure Sensor II with two persons (SSII 2p).

Comparison of the time taken to capture foot, ankle and lower limb morphology is shown in Figure 7. Compared with plaster casting (274.30s ± 33.73s), all 3D scanning methods were significantly faster (*p* < 0.0001), ranging from SSII 2p protocol (26.40s ± 11.09s) to Eva 1p protocol (48.20s ± 16.01s). However, there were no significant differences in the time taken between the four 3D scanning protocols.

## 4 DISCUSSION

The main finding of this study was that the high‐cost Eva and low‐cost SSII 3D scanners using the 1p and 2p protocols and the bespoke scanning jig produced comparable measures of key clinical landmarks compared with cast measures for the fabrication of AFOs in children and adolescents with a movement disorder affecting gait. Some measurements with >20% differences were observed, primarily for rearfoot width, midcalf length and arch height. Future studies should carefully consider the impact of these types of outliers in fabricating AFOs to ensure comfort and fit.

With respect to both percentage difference and Bland and Altman accuracy analyses, the SSII performed slightly better than the Eva, particularly the SSII 2p protocol. However, one patient could not be successfully scanned with the SSII 1p protocol due to involuntary movements, while all reflective markers were captured by the other 3D scanners and protocols for all patients. The four scanning protocols were substantially faster than plaster casting by an expert orthotist, with the SSII 2p protocol being the fastest and Eva 1p protocol being the slowest. Taken together and considering the cost differences of the scanners and workforce implications of the one and two person protocols, the SSII 1p might be considered the preferential approach for the majority of patients requiring AFOs. However, more challenging clinical situations may warrant the 2p protocol with either the SSII or Eva.

The mean bias values from the Bland and Altman plots for the Eva 1p are similar to Desssery and Pallari who reported a mean bias of 2.5 mm (LoA: 17.4–12.5 mm) for the Eva when measuring lower limb circumferences [15]. Although both Eva and SSII 3D scanners use structured light technology, the Eva uses a white light to capture the geometry of the target object and surrounding visible light and heat energy can interfere and disrupt this white light [21]. The SSII, on the other hand, uses infrared light and is hence free from distortion from visible light and heat [22], which might explain the SSII exhibiting slightly better accuracy than the Eva under the study conditions.

Comparing the 1p and 2p protocols, there are several points to consider. For the 1p protocol, the participant may have moved after the clinician‐researcher adjusted the participant's leg into the desired position before starting the 3D scanning procedure. Further, while the 3D scanning jig was used to support the leg, involuntary movements may have occurred during the 3D scanning process. These two factors can introduce motion artifact that can affect the quality of the captured images [23]. Alternatively, in the 2p protocol the foot is held in the desired position by a second clinician‐researcher, which reduces the presence of motion artifact in the 3D scan. However, the 2p protocol results in the hand of the clinician‐researcher appearing in the 3D scan which needs to be digitally removed. The 2p protocol is also the most similar method to plaster casting, as during casting the orthotist often holds the foot and ankle in place or in a corrected position. Interestingly, if the participant's foot is plantarflexed or otherwise out of alignment during the 1p protocol, in silico correction can be applied using specialty software. Certain software designed for fabricating AFOs from a 3D scan may be used to edit the foot position and joint alignment. Noting that software corrections have not been studied or shown to be effective and should be applied with care and avoid correcting foot and ankle joints beyond what physiologically acceptable and tolerated by the patient.

The standardised 3D scanning protocols outlined in this study can be adopted almost immediately in clinical practice and hospital departments or can be used to enhance existing digital workflows. The 3D scanning protocols, including the Scan Stand and the positioning of the patient, could greatly reduce time spent on trial and error for a clinician that could otherwise be spent seeing more patients. Also, reducing the cost of the fabrication method could have a large impact on healthcare provision for countries around the world. Developing countries have difficultly providing adequate care for their population living with disability due to the financial burden of this care. Demonstrating utility of ultra‐low‐cost digital options may lead to more accessible healthcare around the world.

### 4.1 Study limitations

There are some limitations to this study. First, the sample size of patients with movement disorders affecting gait was relatively small which was unavoidable as this PhD project was conducted during the COVID‐19 pandemic. Second, while this study focussed on the utility of 3D scanners compared to plaster casting in terms of accuracy and speed, the patient perspective was not captured. Third, the 3D scan was not used to generate an AFO for a patient, so the fit of the final device was not assessed. In the future, a larger group of pediatric patients with a variety of conditions evaluating patient‐reported outcomes following fabrication of AFOs from 3D scanning protocols should be investigated.

## 5 CONCLUSION

For the fabrication of AFOs in pediatric disorders affecting gait, the high‐cost Eva and low‐cost SSII 3D scanners using the 1p and 2p protocols produced comparable measures of key clinical landmark compared with plaster cast measures and were considerably faster. As the two 3D scanners explored in this study were not significantly different in terms of speed or accuracy, clinicians should consider which additional factors (such as price or portability) may influence their decision in choosing a 3D scanner to implement in their practice.

## AUTHOR CONTRIBUTIONS

**Muhannad Farhan**: Conceptualisation; formal analysis; investigation; methodology; project administration; resources; visualisation; writing—original draft; writing—review & editing. **Joyce Zhanzi Wang**: Investigation; formal analysis; visualisation; writing—review & editing. **Rachael Warncke**: Resources; writing—review & editing. **Tegan Laura Cheng**: Conceptualisation; formal analysis; funding acquisition; methodology; supervision; writing—review & editing. **Joshua Burns**: Conceptualisation; formal analysis; funding acquisition; methodology; supervision; writing—review & editing.

## CONFLICT OF INTEREST STATEMENT

The authors report there are no competing interests to declare.

## ETHICS STATEMENT

All participants that took part in the study signed a written informed consent in accordance with an approved human ethics protocol from the local institution review board (Sydney Children's Hospitals Network Human Research Ethics Committee, protocol 2021/ETH10907).

## CONSENT FOR PUBLICATION

The participants provided written consent for the study and publication of results.

## Supporting information

Supporting Information S1

## Data Availability

The data that support the findings of this study are available on request from the corresponding author. The data are not publicly available due to privacy or ethical restrictions.
