# Supplementary material for: Comparison of accuracy and speed between plaster casting, high‐cost and low‐cost 3D scanners to capture foot, ankle and lower leg morphology of children requiring ankle‐foot orthoses
Source: J Foot Ankle Res. 2024 Aug 27;17(3):e70006. doi: 10.1002/jfa2.70006 (PMC11349604; doi:10.1002/jfa2.70006)
Supplement: Supplementary file 1 — Supporting Information S1 [file JFA2-17-e70006-s001.docx]

## Supplementary files

Supplementary Table 1: 3D scanning protocol for Artec Eva with one person (Eva 1p).

| **Scanning phases** | **Description** |
| --- | --- |
| **Examination table** **position** | The examination table should be positioned in the clinic in a way that ensures there is sufficient space for the operator to move completely around the examination table. |
| **Participant position with the Scan Stand** | The participant should be laying in a prone position on the examination table, with knee flexed at approximately 45° and supported at the shin, foot, and ankle by the 3D scanning jig. The participant is instructed not to move their leg during the 3D scan. |
| **Position of laptop** | Ensure the laptop screen is in the operator’s field of view during the entire scanning progress. |
| **Scanning process**  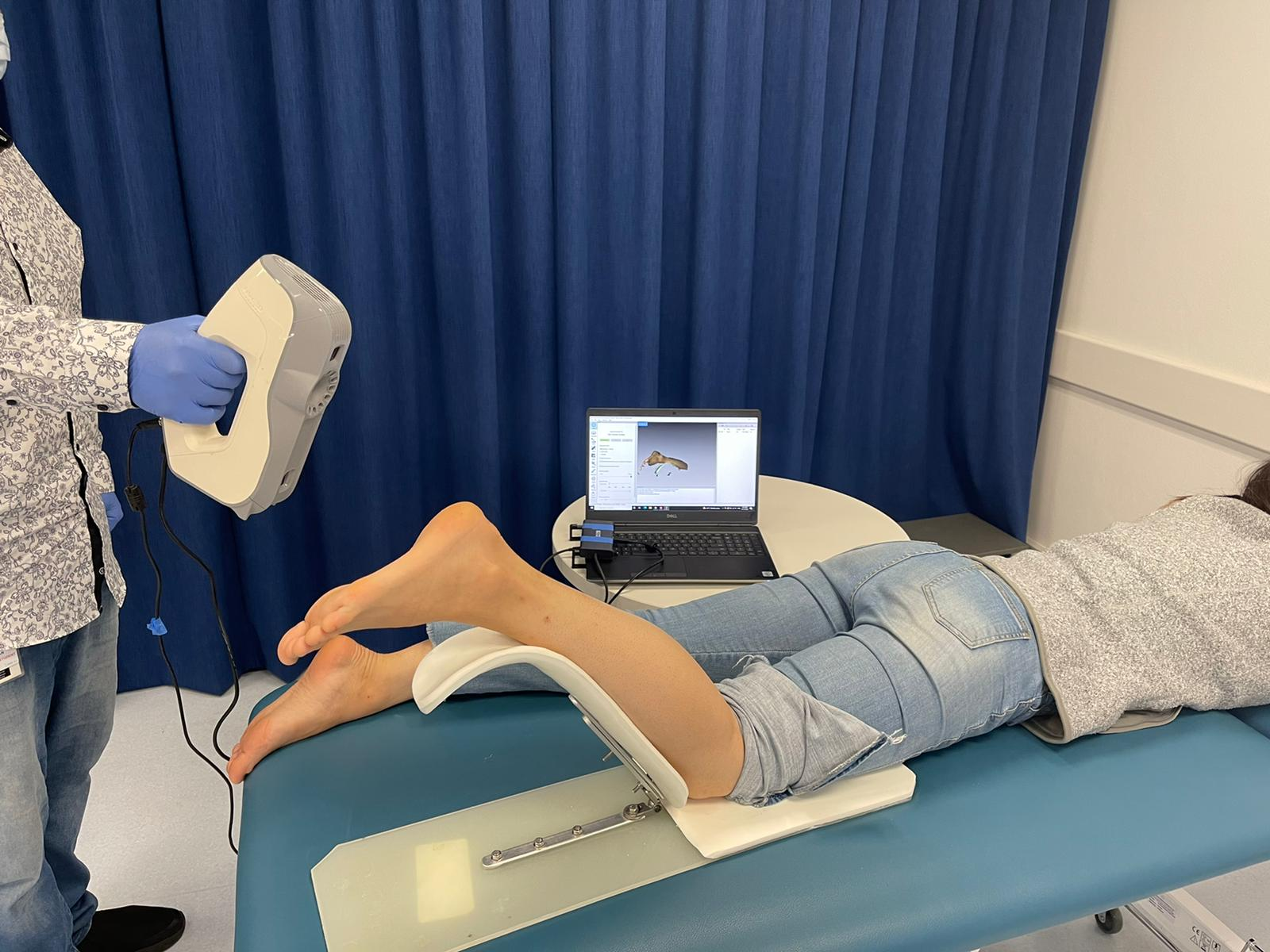 | **Start of scan:** the operator stands at the participant’s side as close to the trunk as comfortable (depending on the room configuration, this can be medial or lateral side) and holds the 3D scanner 40-80 cm from the leg. The 3D scanner should be aimed at the leg at approximately 45° from horizontal, such that the heel and malleolus can be captured in the field of view. The scan can proceed clockwise if starting from the posterior/medial aspect or counter clockwise if starting from the posterior/lateral aspect.  **Mid-scan:** The leg should be 3D scanned using a smooth motion as the operator moves either clockwise or counter clockwise around the patient. Once the operator reaches the opposite side of the trunk from where they started, they continue scanning while moving back to the original starting position, resulting in two sweeps of the lower limb with the 3D scanner. During the scanning process, the operator should ensure that the scan is capturing all relevant surfaces of the foot, ankle and lower leg using the second sweep to fill in any missing areas.  **End of scan:** the operator should aim to end the scan at the same location they started. Ideally, the scan should be stopped while still aiming at the participant to reduce artefact capture. |
| **Processing and exporting scan** | Use Artec Studio software to process the scan and export the mesh in a compatible file format (e.g. stl or ply) |

Supplementary Table 2: 3D scanning protocol for Artec Eva with two persons (Eva 2p).

| **Scanning steps** | **Description** |
| --- | --- |
| **Examination table** **position** | The examination table should be positioned in the clinic in a way that ensures there is sufficient space for the operator to move completely around the examination table. |
| **Participant position with the Scan Stand** | The participant should be laying in a prone position on the examination table, with knee flexed at approximately 45° and supported at the shin, foot, and ankle by the 3D scanning jig. The participant is instructed not to move their leg during the 3D scan. |
| **Position of laptop** | Ensure the laptop screen is in the scanning operator’s field of view during the entire scanning progress. |
| **Scanning process**  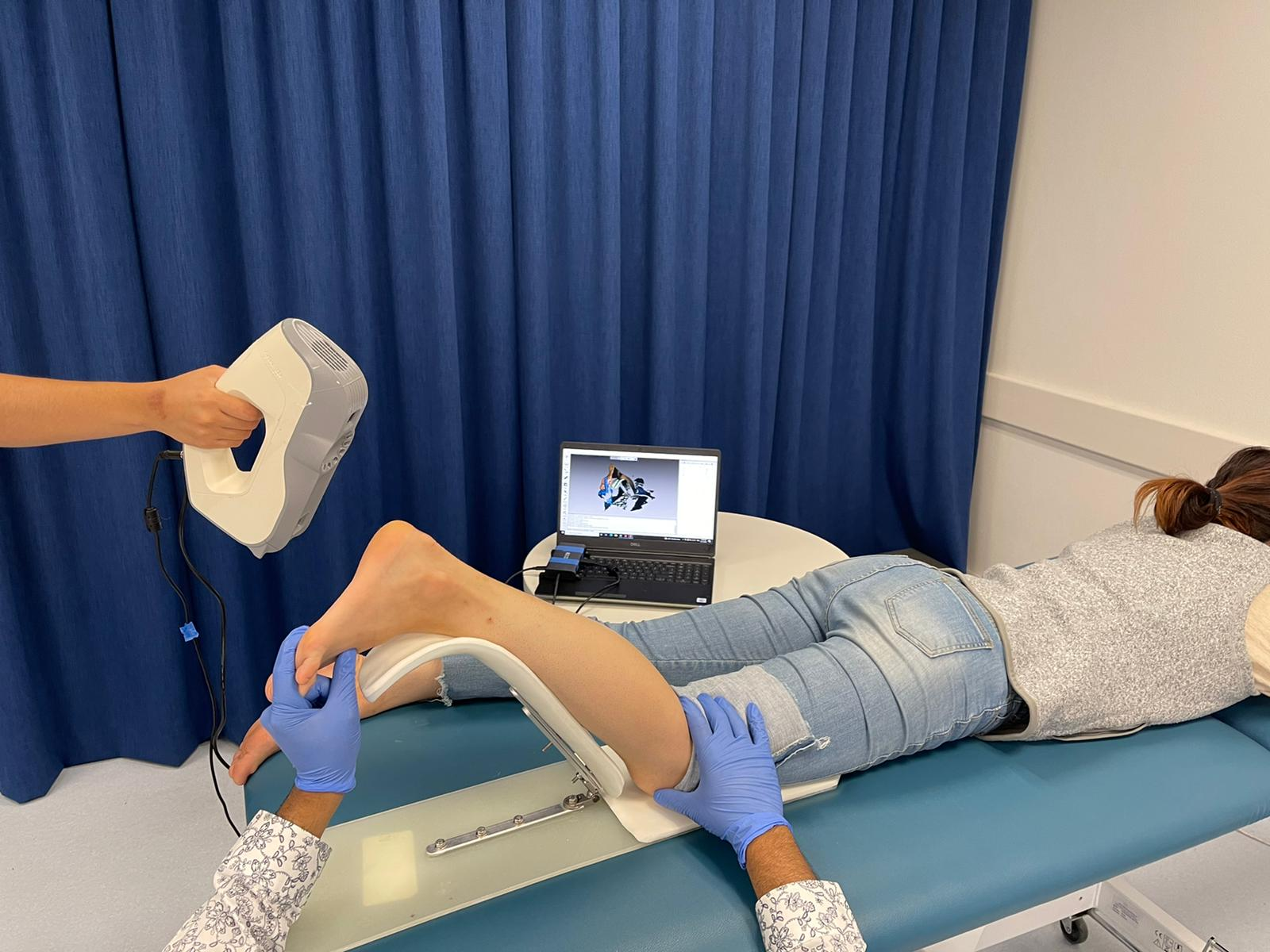 | **First operator:**  Holds the participant’s foot in as neutral a position as possible. Squatting next to the examination table can assist in staying out of the 3D scanning field.  **Second operator:**  **Start of scan:** the operator stands at the participant’s side as close to the trunk as comfortable (depending on the room configuration, this can be medial or lateral side) and holds the 3D scanner 40-80 cm from the leg. The 3D scanner should be aimed at the leg at approximately 45° from horizontal, such that the heel and malleolus can be captured in the field of view. The scan can proceed clockwise if starting from the posterior/medial aspect or counter clockwise if starting from the posterior/lateral aspect.  **Mid-scan:** The leg should be 3D scanned using a smooth motion as the operator moves either clockwise or counter clockwise around the participant. Once the operator reaches the opposite side of the trunk from where they started, they continue scanning while moving back to the original starting position, resulting in two sweeps of the lower limb with the 3D scanner. During the scanning process, the operator should ensure that the scan is capturing all relevant surfaces of the foot, ankle and lower leg using the second sweep to fill in any missing areas.  **End of scan:** the operator should aim to end the scan at the same location they started. Ideally, the scan should be stopped while still aiming at the participant to reduce artefact capture. |
| **Processing and exporting scan** | Use Artec Studio software to process the scan and export the mesh in a compatible file format (e.g., stl or ply) |

Supplementary Table 3: 3D scanning protocol for Structure Sensor Mark II with one person (SSII 1p).

| **Scanning steps** | **Description** |
| --- | --- |
| **Examination table** **position** | The examination table should be positioned in the clinic in a way that ensures there is sufficient space for the operator to move completely around the examination table. |
| **Participant position with the Scan Stand** | The participant should be laying in a prone position on the examination table, with knee flexed at approximately 45° and supported at the shin, foot, and ankle by the 3D scanning jig. The participant is instructed not to move their leg during the 3D scan. |
| **Scanning process**  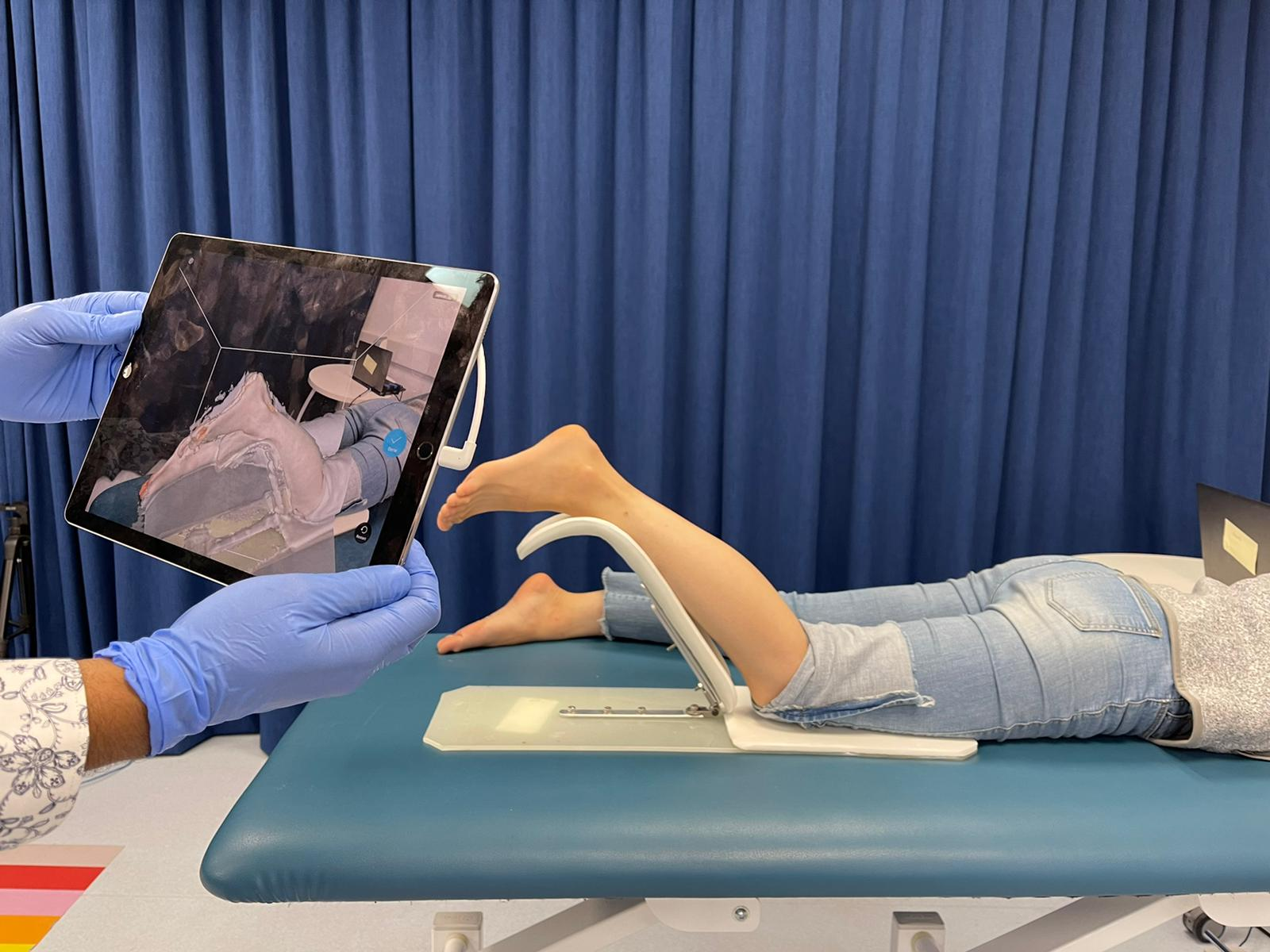 | **Start of scan:** the operator stands at the participant’s side as close to the trunk as comfortable (depending on the room configuration, this can be medial or lateral side) and holds the 3D scanner 30-100 cm from the leg, ensuring the leg is inside the red box. The 3D scanner should be aimed at the leg at approximately 45° from horizontal, such that the heel and malleolus can be captured in the field of view. The scan can proceed clockwise if starting from the posterior/medial aspect or counter clockwise if starting from the posterior/lateral aspect.  **Mid-scan:** The leg should be 3D scanned using a smooth motion as the operator moves either clockwise or counter clockwise around the participant. Once the operator reaches the opposite side of the trunk from where they started, they continue scanning while moving back to the original starting position, resulting in two sweeps of the lower limb with the 3D scanner. During the scanning process, the operator should ensure that the scan is capturing all relevant surfaces of the foot, ankle and lower leg using the second sweep to fill in any missing areas.  **End of scan:** the operator should aim to end the scan at the same location they started. Ideally, the scan should be stopped while still aiming at the participant to reduce artefact capture. |
| **Processing and exporting scan** | Use the automated file processing and export once complete |

Supplementary Table 4: 3D scanning protocol for Structure Sensor Mark II with two persons (SSII 2p).

| **Scanning steps** | **Description** |
| --- | --- |
| **Examination table** **position** | The examination table should be positioned in the clinic in a way that ensures there is sufficient space for the operator to move completely around the examination table. |
| **Participant position with the Scan Stand** | The participant should be laying in a prone position on the examination table, with knee flexed at approximately 45° and supported at the shin, foot, and ankle by the 3D scanning jig. The participant is instructed not to move their leg during the 3D scan. |
| **Scanning process**  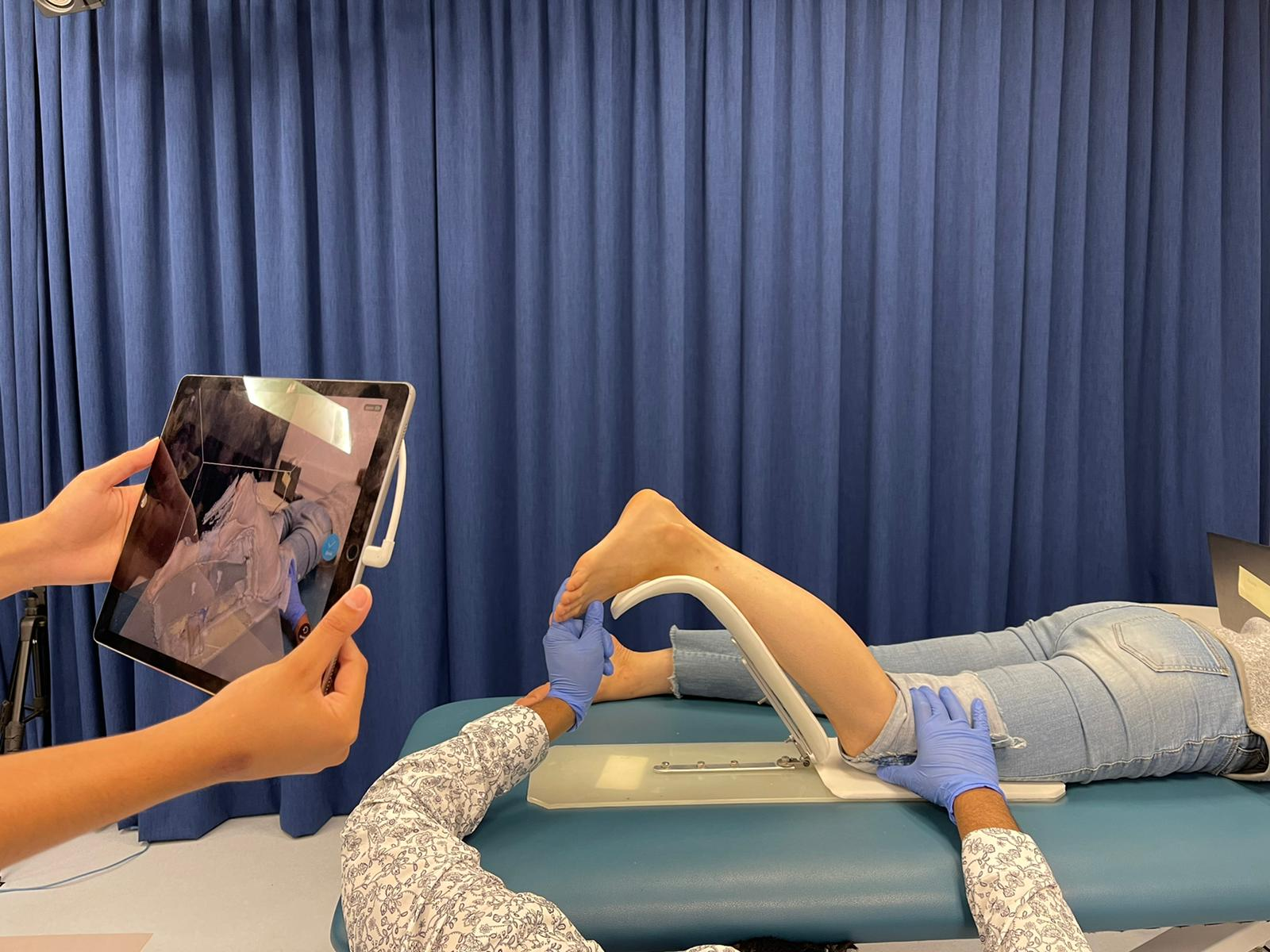 | **First operator:**  Holds the participant’s foot in as neutral a position as possible. Squatting next to the examination table can assist in staying out of the 3D scanning field.  **Second operator:**  **Start of scan:** the operator stands at the participant’s side as close to the trunk as comfortable (depending on the room configuration, this can be medial or lateral side) and holds the 3D scanner 30-100 cm from the leg, ensuring the leg is inside the red box. The 3D scanner should be aimed at the leg at approximately 45° from horizontal, such that the heel and malleolus can be captured in the field of view. The scan can proceed clockwise if starting from the posterior/medial aspect or counter clockwise if starting from the posterior/lateral aspect.  **Mid-scan:** The leg should be 3D scanned using a smooth motion as the operator moves either clockwise or counter clockwise around the participant. Once the operator reaches the opposite side of the trunk from where they started, they continue scanning while moving back to the original starting position, resulting in two sweeps of the lower limb with the 3D scanner. During the scanning process, the operator should ensure that the scan is capturing all relevant surfaces of the foot, ankle and lower leg using the second sweep to fill in any missing areas.  **End of scan:** the operator should aim to end the scan at the same location they started. Ideally, the scan should be stopped while still aiming at the participant to reduce artefact capture. |
| **Processing and exporting scan** | Use the automated file processing and export once complete |

Supplementary Table 5: The clinical measurements of the participants

| Participant | Parameter | Measurement (mm) |
| --- | --- | --- |
| 1 | Forefoot | 56.9 |
|  | Rearfoot | 48.9 |
|  | Malleolus | 64.5 |
|  | Midcalf | 81.3 |
|  | Foot length | 170 |
|  | Arch height | 37.2 |
| 2 | Forefoot | 80.7 |
|  | Rearfoot | 52.3 |
|  | Malleolus | 66.4 |
|  | Midcalf | 100.2 |
|  | Foot length | 190.3 |
|  | Arch height | 46 |
| 3 | Forefoot | 71.5 |
|  | Rearfoot | 43.5 |
|  | Malleolus | 61.7 |
|  | Midcalf | 85.3 |
|  | Foot length | 181 |
|  | Arch height | 43 |
| 4 | Forefoot | 81.1 |
|  | Rearfoot | 50.6 |
|  | Malleolus | 62.4 |
|  | Midcalf | 79.4 |
|  | Foot length | 190.6 |
|  | Arch height | 37.3 |
| 5 | Forefoot | 89.7 |
|  | Rearfoot | 58.8 |
|  | Malleolus | 76.1 |
|  | Midcalf | 91.9 |
|  | Foot length | 240.5 |
|  | Arch height | 38 |
| 6 | Forefoot | 88.6 |
|  | Rearfoot | 59.9 |
|  | Malleolus | 72.3 |
|  | Midcalf | 89.7 |
|  | Foot length | 221.5 |
|  | Arch height | 51.5 |
| 7 | Forefoot | 78.6 |
|  | Rearfoot | 56.3 |
|  | Malleolus | 58.8 |
|  | Midcalf | 95.2 |
|  | Foot length | 198.1 |
|  | Arch height | 33.1 |
| 8 | Forefoot | 76.9 |
|  | Rearfoot | 49 |
|  | Malleolus | 54.6 |
|  | Midcalf | 64.2 |
|  | Foot length | 185 |
|  | Arch height | 36 |
| 9 | Forefoot | 80 |
|  | Rearfoot | 52 |
|  | Malleolus | 61.2 |
|  | Midcalf | 79.2 |
|  | Foot length | 205 |
|  | Arch height | 37.5 |
| 10 | Forefoot | 78 |
|  | Rearfoot | 54.5 |
|  | Malleolus | 71.8 |
|  | Midcalf | 84.2 |
|  | Foot length | 210.5 |
|  | Arch height | 48.8 |

Supplementary Figure 1: Bland and Altman plots illustrating agreement between 3D scan and casts measures for forefoot width. The 3D scanning protocols are Artec Eva with one person (Eva 1p), Artec Eva with two persons (Eva 1p), Structure Sensor II with one person (SSII 1p), and Structure Sensor II with two persons (SSII 2p).

Supplementary Figure 2: Bland and Altman plots illustrating agreement between 3D scan and casts measures for rearfoot width. The 3D scanning protocols are Artec Eva with one person (Eva 1p), Artec Eva with two persons (Eva 1p), Structure Sensor II with one person (SSII 1p), and Structure Sensor II with two persons (SSII 2p).

Supplementary Figure 3: Bland and Altman plots illustrating agreement between 3D scan and casts measures for malleoli width. The 3D scanning protocols are Artec Eva with one person (Eva 1p), Artec Eva with two persons (Eva 1p), Structure Sensor II with one person (SSII 1p), and Structure Sensor II with two persons (SSII 2p).

Supplementary Figure 4: Bland and Altman plots illustrating agreement between 3D scan and casts measures for midcalf width. The 3D scanning protocols are Artec Eva with one person (Eva 1p), Artec Eva with two persons (Eva 1p), Structure Sensor II with one person (SSII 1p), and Structure Sensor II with two persons (SSII 2p).

Supplementary Figure 5: Bland and Altman plots illustrating agreement between 3D scan and casts measures for foot length. The 3D scanning protocols are Artec Eva with one person (Eva 1p), Artec Eva with two persons (Eva 1p), Structure Sensor II with one person (SSII 1p), and Structure Sensor II with two persons (SSII 2p).

Supplementary Figure 6: Bland and Altman plots illustrating agreement between 3D scan and casts measures for arch height. The 3D scanning protocols are Artec Eva with one person (Eva 1p), Artec Eva with two persons (Eva 1p), Structure Sensor II with one person (SSII 1p), and Structure Sensor II with two persons (SSII 2p).
